# Urbanization, environment, and inflammaging: insights from sub-Saharan Africa

**DOI:** 10.1038/s41514-026-00362-0

**Published:** 2026-03-13

**Authors:** Stephen W. Bickler, Matchecane Cossa, Irina Mendes de Sousa, Emília V. Noormahomed

**Affiliations:** 1 Division of Pediatric Surgery, Department of Surgery, UC San Diego, San Diego, CA USA; 2 Department of Surgery, Faculty of Medicine, Eduardo Mondlane University (UEM), Maputo, Mozambique; 3 Ministry of Health, Maputo, Mozambique; 4 Mozambique Institute for Health Education and Research (MIHER), Maputo, Mozambique; 5 Department of Biological Sciences, Faculty of Sciences, Eduardo Mondlane University (UEM), Maputo, Mozambique; 6 Department of Microbiology, Faculty of Medicine, Eduardo Mondlane University (UEM), Maputo, Mozambique

**Keywords:** Biomarkers, Diseases, Immunology

## Abstract

Emerging evidence shows that inflammaging varies across populations, challenging universal immune-aging models. Urbanization in sub-Saharan Africa—characterized by reduced exposure to infectious diseases and rising rates of noncommunicable diseases—offers a natural experiment for assessing environmental effects on inflammaging. Lower inflammaging in indigenous groups may reflect adaptation to chronic infection, whereas heightened inflammation in industrialized populations suggests ecological imbalance, underscoring the need to include diverse ecological groups in aging research.

## Introduction

A growing body of research comparing industrialized and indigenous populations shows that inflammaging—often thought of as a universal hallmark of aging—is far from consistent across human environments^1–3^. Studies of cytokine axis structure in groups such as the Tsimane and Orang Asli reveal little evidence of the classic inflammaging pattern seen in high-income countries, suggesting that chronic age-related inflammation is largely a product of industrialized lifestyles rather than an inevitable biological process. Findings from rural–urban comparisons demonstrate that, while some markers such as interleukin-6 (IL-6) may rise modestly with age, broader proinflammatory profiles do not consistently increase in non-industrialized settings, and in some cases only emerge with lifestyle change^3^. Together, this work challenges the presumed universality of the inflammaging model and highlights the need to better understand how environmental factors influence health and disease. Foremost, it raises a fundamental question: how can the consequences of inflammation vary so markedly across different environments? In indigenous populations, inflammation is a critical component of the immune response, conferring survival benefits by protecting against infectious diseases. By contrast, in industrialized settings, chronic low-grade inflammation is thought to be maladaptive, driving the pathogenesis of many age-related diseases^4^. This clear difference implies that the immune system must possess flexible regulatory mechanisms that fine-tune inflammatory responses, maintaining a state that is “just right” for a given ecological niche.

## Urbanization in sub-Saharan Africa as a natural experiment

In considering how inflammaging is shaped by the environment, important lessons can be drawn from the biological changes that occur with urbanization in sub-Saharan Africa (SSA). SSA offers a unique perspective on this problem because urbanization is occurring rapidly, and there are often extreme differences in the burden of infectious diseases between rural and urban areas due to variations in socioeconomic development (for review, see Bickler et al.^5^). A further advantage of studying urbanization in Africa is that these studies can be done in genetically homogenous populations. Urbanization also produces distinct epidemiological profiles: communicable diseases predominate in rural areas, while urban populations increasingly exhibit a higher prevalence of noncommunicable diseases (NCDs)^6,7^. This epidemiological transition mirrors broader global trends, where NCDs such as diabetes, atherosclerosis, and cancers are now the leading cause of death^8,9^.

## Early-life infection, environmental enteropathy, and immune adaptation

Like the Tsimane from the Bolivian Amazon and the Orang Asli from Peninsular Malaysia, rural populations in SSA experience a heavy burden of chronic and recurrent infections that begin early in life. Much of this infection centers on the gut because of poor waste disposal and limited access to safe drinking water. Notably, environmental enteropathy (EE), also known as Environmental Enteric Dysfunction, is highly prevalent in these environments. EE is an enigmatic condition of the small bowel characterized by loss of barrier function, bacterial overgrowth in the small intestine, malabsorption, and low-grade intestinal inflammation leading to small intestinal villous atrophy^10^. Children with EE exhibit increased levels of serum endotoxin and anti-endotoxin antibodies, which correlate with the severity of enteropathy and the degree of growth failure^11^. Additionally, there is systemic inflammation, as indicated by elevated white blood cell counts and C-reactive protein levels. This picture is further complicated by the near-ubiquitous exposure to parasitic infections, including malaria, Schistosomiasis, and soil-transmitted helminths. The biological consequences of this heavy burden of infectious diseases are considerable: undernutrition is widespread, growth faltering is common, and under-five mortality rates approach 100 deaths per 1000 live births in the poorest countries.

For those children who survive this barrage of infectious diseases early in life, their immune systems are permanently altered. This “immunological adaptation” to chronic and recurrent infection has been aptly described by Greenwood and Whittle^12^:

“A peasant farmer can carry out a full day’s work in the fields and lives a normal social life is considered a ‘healthy normal’ in many developing communities. However, by the medical standards of industrialized countries, he would be considered far from healthy. He is likely to have had repeated attacks of acute malaria since early childhood and still have episodes of asymptomatic malaria parasitemia. He may well have Schistosomiasis, although now this produces little in the way of symptoms, and he probably has two or three types of intestinal worms. He may have asymptomatic filariasis. In addition, he is likely to have passed through a period of malnutrition at the time of weaning, which may have had permanent effects on the immune system. Such a ‘healthy’ normal shows immunological effects on the immunological changes that are not found among protected individuals in industrialized societies.”

## Hypothesis: urbanization unmasks proinflammatory pathways

Our working hypothesis is that chronic and recurrent infections dampen proinflammatory signaling pathways in rural areas^5^. We focus on chronic and recurrent infections because pathogens are the leading cause of death in these settings. When rural populations transition to urban lifestyles, pathogen exposure diminishes, driven by improvements in sanitation, access to clean water, and better healthcare. With this reduction in the burden of infectious diseases, the suppressive forces that previously kept proinflammatory pathways in check are released, unmasking underlying immune circuits and contributing to rising rates of inflammation-driven NCDs such as atherosclerosis, cancer, and autoimmune disorders. From this perspective, the absence of an inflammaging axis in the Tsimane and Orang Asli should not be interpreted as immunological deficiency, but rather as immunological adaptations that confer a survival advantage. Conversely, the exaggerated inflammaging observed in industrialized settings may represent an immune system that has lost its long-standing environmental counterbalance and has shifted to a proinflammatory state. Our hypothesis is well aligned with the general principles of the Hygiene and Old Friends hypotheses. The Hygiene Hypothesis suggests that the level of sanitation affects the risk of Western diseases such as appendicitis and atopic diseases^13,14^. The Old Friends Hypothesis postulates that exposure to microorganisms with which humans co-evolved is an essential driver of the regulatory and anti-inflammatory arm of the immune system^15^.

## Possible mechanisms linking the environment to inflammaging

One of the most compelling examples of environmentally driven immune modulation comes from ex vivo cytokine stimulation studies. Temba and colleagues performed whole-blood assays in rural and urban Tanzanian adults, revealing elevated production of proinflammatory cytokines—TNF-α, IFN-γ, and IL-6—in urban residents compared to their rural counterparts following an immune challenge^16^. To explain this heightened cytokine responsiveness, we have drawn on mechanistic insights from studies of rheumatoid arthritis, where a “TNF-hypersecreting” phenotype has been linked to impaired mitochondrial aspartate synthesis, reduced NAD⁺ regeneration, and heightened endoplasmic reticulum (ER) stress^17^. Together, these metabolic disturbances converge to promote TNF-α overproduction. We have suggested this mechanism might also, in part, explain the increased production of proinflammatory cytokines in urban compared to rural populations in SSA^18^. Support for this hypothesis comes from untargeted metabolomics studies in Tanzania, which show that rural individuals have higher levels of aspartate and other TCA cycle intermediates (succinate, pyruvate, citrate, and L-glutamate) in both serum and stool, compared to urban cohorts. These metabolic profiles coincide with lower TNF-α and IL-6 responses following ex vivo immune stimulation. Conversely, urban dwellers exhibit lower serum and stool aspartate levels and heightened proinflammatory cytokine production.

More broadly, we have proposed a role for mitochondria in mediating the immunological and metabolic changes that occur with urbanization^19^. Using transcriptomic data from peripheral blood leukocytes of rural and urban Moroccan adults, we observed that 76.5% of nuclear-encoded mitochondrial genes were differentially expressed, with 97% upregulated in urban dwellers. Enrichment analysis identified 367 significantly enriched pathways, with oxidative phosphorylation, insulin secretion, and glucose regulation being the top three. Mitochondrial RNA metabolic processing and translational elongation were the biological processes that had the greatest enrichment. Of note, several other gene expression studies in Africa have also reported enrichment of the oxidative phosphorylation pathway in urban areas^16,20^. The importance here is that changes in mitochondrial function could offer a mechanistic bridge between environmental change and inflammaging. Mitochondria act as metabolic hubs and immune signal transducers, and their dysfunction has been implicated in a wide range of inflammatory and neurodegenerative diseases. However, because changes in gene expression do not necessarily translate into changes in mitochondrial function, further studies are needed to better define the role mitochondria play in the biological changes that occur with urbanization.

## Implications, knowledge gaps, and future directions

Industrialization clearly reshapes immune function, yet our knowledge of how it remodels the immune, metabolic, and microbial systems remains incomplete. Critical questions persist regarding the complex interactions between immunity and metabolism^21–24^ and how dietary shifts toward processed foods, refined sugars, and novel additives alter these pathways. Equally important will be clarifying how changes in the intestinal microbiome during urbanization influence inflammatory and metabolic regulation. Industrialization dramatically alters the intestinal microbiome by reducing diversity and shifting microbial composition^25–27^. Distinguishing pathogen-mediated effects from those driven by sedentary behavior, diet, smoking, and other lifestyle factors will require more targeted research, particularly in populations undergoing rapid ecological transition.

In conclusion, the rural–urban transition in SSA offers a unique opportunity to study how environmental factors influence the inflammaging process. A longitudinal cohort study tracking changes in an individual’s transcriptome, proteome, and metabolome would be the ideal approach for revealing how chronic and recurrent infection, lifestyle change, and environmental exposures jointly shape inflammaging. Ultimately, advancing this work will deepen our understanding of NCD pathogenesis and challenge assumptions about the inevitability of immune decline with age. Most importantly, it reinforces the need to include ethnically and ecologically diverse populations in immunological research to build a more comprehensive and accurate model of immune aging.

## Data Availability

No new data.
